# Supplementary material for: Effect of chronic kidney disease on outcomes following proximal humerus fragility fracture surgery in diabetic patients: A nationwide population-based cohort study
Source: PLoS One. 2021 Oct 8;16(10):e0258393. doi: 10.1371/journal.pone.0258393 (PMC8500432; doi:10.1371/journal.pone.0258393)
Supplement: S1 Table — (DOCX) [file pone.0258393.s001.docx]

**S1 Table.** ICD-9-CM code used for diagnosis in the study

| Variable | ICD-9-CM code |
| --- | --- |
| Diabetes mellitus type 2 | 250.xx excluding type 1 DM |
| Diabetes mellitus type 1 | 250.01, 250.03, 250.11, 250.13, 250.21, 250.23, 250.31, 250.33, 250.41, 250.43, 250.51, 250.53, 250.61, 250.63, 250.71, 250.73, 250.81, 250.83, 250.91, 250.93 |
| Chronic kidney disease | 580.xx–589.xx, 403.xx–404.xx, 016.0x, 095.4x, 236.9x, 250.4x, 274.1x, 442.1x, 447.3x, 440.1x, 572.4x, 642.1x, 646.2x, 753.1x, 283.11, 403.01, 404.02, 446.21 |
| Dialysis | 585.xx (Catastrophic illness card) |
| Malignancy | 140.xx–208.xx (Catastrophic illness card) |
| Multiple trauma | 959.99 |
| Immune diseases | 710.0, 710.1, 714.0, 710.4, 710.3, 446.0, 446.2, 446.4, 446.5, 443.1, 446.7, 136.1, 694.4, 710.2, 555.xx, 556.xx, 714.30–714.33 |
| Implant infection | 996.66, 996.67 |
| Chronic obstructive pulmonary disease | 491.xx, 492.xx, 496.xx |
| Heart failure | 428.xx |
| Coronary heart disease | 410.xx–414.xx |
| Hyperlipidemia | 272.xx |
| Cardiac dysrhythmia | 427.xx |
| Old myocardial infarction | 410.xx, 412.xx |
| Hypertension | 401.xx–405.xx |
| Stroke | 430.xx–432.xx, 433.xx–437.xx |
| Dementia | 290.xx, 294.xx |
| Osteoporosis | 733.xx |

ICD-9-CM, International Classification of Diseases, Ninth Revision, Clinical Modification.
