# Supplementary material for: Effect of chronic kidney disease on outcomes following proximal humerus fragility fracture surgery in diabetic patients: A nationwide population-based cohort study
Source: PLoS One. 2021 Oct 8;16(10):e0258393. doi: 10.1371/journal.pone.0258393 (PMC8500432; doi:10.1371/journal.pone.0258393)
Supplement: Attachment — Submitted filename: Response to reviewers.docx [file pone.0258393.s002.docx]

**Dear editor:**

**Thank you** for the nice and detailed review. We've provided a **point-by-point revision** to all of your comments. The reasons and revisions are provided in the following tables. In the revised manuscript, all the changes are highlighted in **red.** We deeply appreciate your valuable operations, which stimulated a more thorough consideration of the essay. Thank you.

Sincerely

**Liang-Tseng Kuo**

**Aug 20^th^, 2021**

**Reviewer 1**

| **Opinion** | **Reply** |
| --- | --- |
| Comments to the Author:  Reviewer #1: Good review of cases of Proximal Humerus Fracture correlated to CRF and DM.  Possible to change the recommendation of treatment for this group of patient with high risk, from surgical to non-surgical treatment. | Response:  Thanks for your comments. We did point-to-point responses to your comments in the following sections. |

**Reviewer 2**

| **Opinion** | **Reply** |
| --- | --- |
| Comments to the Author:  Reviewer #2: This is an interesting stud. A few comments for revision: | Response:  Thanks for your comments. We did point-to-point responses to your comments in the following sections. |
| 1. In Figures 2A-D: replace "normal" with non-CKD | Response 1:  Thanks for your comments. We corrected all typos, and replaced normal with non-CKD. |
| 2. Line 272 – what population is this referring to? Within dialysis-requiring CKD? | Response 2:  Thanks for your comments. This referred to dialysis-requiring CKD group. We rephrased this sentence to make it clearer as following:  **Line 272**  Advanced age, poor compliance, and comorbidities have been validated as predictors of admission for causes other than vascular access related in dialysis patients. |
| 3. Line 276 – What does “this cohort” refer to – the whole registry, or your cohort of diabetes subjects? (given that, in line 279 you then list another set of % of mortality “in our cohort”) | Response 3:  Thanks for your great opinion. This cohort referred to the diabetic patients in the current study. Therefore, we revised these two sentences to make it clearer for readers:  **Line 276**  The one-year mortality in the current study was about 5 %, similar to that previously reported for the general population's proximal humerus fracture  **Line 279**  In this study, 8.9% of CKD patients and 18.9% of dialysis patients died one year after surgery. |
| 4. Line 290 – higher or lower mortality risk in upper arm fractures? | Response 4:  Thanks for your opinion. Mandai et al. showed that upper arm fracture was had a higher odd of death than hip fracture when comparing to leg fracture (upper arm fracture, odds ratio (OR)= 2.01, 95% confidence interval (CI), 1.01 to 4.01; hip fracture, OR=1.77, 0.98 to 3.18). This finding was different from that in the current study. We revised this paragraph as following:  **Line 288**  Mandai et al.[35] demonstrated that upper arm fracture was associated with 2-times higher odds of vascular access failure in dialysis patients. Therefore, dialysis patients with upper arm fractures had a slightly higher 30-day mortality risk compared to those with hip fractures. |
| 5. Line 320 – suggest adding in “in diabetes patients” in first sentence | Response 5:  Thanks for your comments. We added “in diabetic patients” in the end of this sentence, and revised as following:  **Line 320**  Deteriorated renal function is associated with elevated risks for infection, revision, readmission, and mortality after proximal humerus fracture surgery in diabetic patients. |

**Journal Office**

| **Opinion** | **Reply** |
| --- | --- |
|  | We have revised all necessary part as the request of reviewers and re-uploaded the revised manuscript. |
